# Nanostructured Carbon-Doped BN for CO_2_ Capture Applications

**DOI:** 10.3390/nano13172389

**Published:** 2023-08-22

**Authors:** Rimeh Mighri, Kevin Turani-I-Belloto, Umit B. Demirci, Johan G. Alauzun

**Affiliations:** 1 Institut Charles Gerhardt, Univ Montpellier, CNRS, ENSCM, 34095 Montpellier, France; 2 Institut Europeen des Membranes, IEM—UMR 5635, Univ Montpellier, ENSCM, CNRS, 34095 Montpellier, France

**Keywords:** boron nitride, carbon-doped, ammonia borane, alkyl amine borane adduct, microporosity, CO_2_ capture

## Abstract

Carbon-doped boron nitride (denoted by BN/C) was prepared through the pyrolysis at 1100 °C of a nanostructured mixture of an alkyl amine borane adduct and ammonia borane. The alkyl amine borane adduct acts as a soft template to obtain nanospheres. This bottom-up approach for the synthesis of nanostructured BN/C is relatively simple and compelling. It allows the structure obtained during the emulsion process to be kept. The final BN/C materials are microporous, with interconnected pores in the nanometer range (0.8 nm), a large specific surface area of up to 767 m^2^·g^−1^ and a pore volume of 0.32 cm^3^·g^−1^. The gas sorption studied with CO_2_ demonstrated an appealing uptake of 3.43 mmol·g^−1^ at 0 °C, a high CO_2_/N_2_ selectivity (21) and 99% recyclability after up to five adsorption–desorption cycles.

## 1. Introduction

In recent years, numerous studies have been carried out regarding the purification and storage of gas. For environmental issues, the most studied one is carbon dioxide, or CO_2_ [1]. Due to its simplicity, low energy consumption and ease of reusability, gas capture on a solid material seems the most promising. For this purpose of gas purification and/or storage (CO_2_, H_2_, O_2_, etc.), many materials have been developed. They must combine many characteristics: a specific affinity or selectivity toward the target component, recyclability, a low cost and, of course, a high adsorption uptake. In addition, these materials should have a good tolerance for relatively harsh conditions.

The type of materials considered is extremely vast, but porous materials tend to be the most interesting [2]. It is indeed possible to control the composition (oxides, hybrids, MOFs, zeolites or ceramics), structure (crystalline or amorphous), texture and porosity (micro-, meso- or macro-) of such materials [3,4,5].

We recently presented a series of porous B_x_C_y_N_z_ materials synthesized from ethane 1,2 diamineborane (EDAB) using a porosity agent (F-127). These BCN materials were mainly microporous with specific surface areas of up to 510 m^2^·g^−1^ and a pore volume of 0.35 cm^3^·g^−1^. We have demonstrated that these materials have potential for CO_2_ capture. Indeed, they showed a good CO_2_ uptake of up to 3.23 mmol·g^−1^ at 0 °C as well as a remarkable CO_2_/N_2_ selectivity value of 26 (evaluated using the IAST method) [6].

Among all porous materials with potential applications for gas adsorption, boron nitride (BN), with its outstanding thermal and chemical stability, might be of great interest. BN can indeed be used in applications within corrosive environments where the widely investigated oxide ceramics cannot work properly. Boron nitride (BN) is a non-oxide ceramic with a very wide range of applications, such as for energy or medicine [7,8,9]. It can be synthesized from numerous molecular precursors, and notably, from ammonia borane (AB) [9,10,11]. AB is a molecule with the chemical structure H_3_N-BH_3_. Pristine AB is a crystalline, stable solid at room temperature and under an inert atmosphere. These characteristics are mainly related to the dihydrogen bonds H^δ+^···H^δ−^ occurring between molecules. Due to its high gravimetric hydrogen density (19.6% by weight), AB has been widely studied in recent decades for the chemical storage of hydrogen. It has been demonstrated that the thermolysis of AB (in three steps), releasing one equivalent of H_2_ at each step, leads to the formation of crystalline hexagonal BN (h-BN) between 1200 and 1400 °C [6].

In the field of gas purification and storage, particularly for CO_2_, BN appears to be an interesting material due to its properties [12]. Computer calculations have predicted strong interactions between CO_2_ and BN nanotubes under ambient conditions (with a physisorption energy of 0.21 eV and a chemisorption energy of 0.82 eV). Several experimental studies have already reported the adsorption of CO_2_ by different BN structures, a few of which involved porous BN for selective CO_2_ adsorption under conditions close to those of the environment [13,14,15,16,17]. One example is a porous BN structure modified by a surface-active copolymer (P123) [16] with a specific surface area of 476 m^2^·g^−1^. This material was able to capture 2.68 mmol·g^−1^ (or 118.3 mg·g^−1^) of CO_2_ in 6 h, at 25 °C and under a constant gas flow (50 mL·min^−1^).

We also believe BN-based material to be a valuable alternative to other porous materials for the removal of exhaust gas pollutants. Unfortunately, many steps are needed to synthesize porous BN materials with a good composition, structure and porosity (such as hard templating methods or polymer-derived ceramic routes) [18,19]. Moreover, we suspect that defects within the BN structure and/or carbon-doping would be needed to improve gas sorption [9]. In the present work, a porous carbon-doped BN is synthesized in order to enhance its CO_2_ adsorption performance.

In order to synthesize porous BN, we intended to nanosize AB by adopting methods recently presented [20]. In a previous work, Lai et al. nanosized AB particles using soft templates (surfactants) instead of a hard template agent [21]. They firstly chose oleic acid, C_18_H_34_COOH, for this purpose. The AB nanoparticles were obtained using the antiprecipitation method, whereby a tetrahydrofuran solution of AB kept at 45 °C was added to a cyclohexane solution of oleic acid kept at 15 °C. A white precipitate consisting of AB nanoparticles with a diameter of ca. 50 nm was collected. In a second work, cetyltrimethylammonium bromide, C_16_H_33_N(CH_3_)_3_Br, was used [20]. It was shown to be effective as a common oxygen-free soft template to allow the self-assembly of AB nanoparticles. These AB nanoparticles, with an average dimeter of 110 nm, were prepared by the emulsification method using dodecane, C_12_H_26_, as a counter-solvent. These two seminal works have paved the way for new research on AB, and more broadly, on ammonia borane adducts (ABAs) and soft templates made only of boron, nitrogen, carbon and hydrogen (free of, e.g., oxygen and halides).

In order to pursue the structuration studies of AB leading to BN materials, alkyl amine borane adducts (ABAs) were used as soft templating agents. We have previously described the synthesis of primary alkylamine borane adducts, C_x_H_2x+1_NH_2_BH_3_ (CxAB), with butyl, hexyl, octyl, decyl, dodecyl, tetradecyl, hexadecyl and octadecyl chains (x = 4, 6, 8, 10, 12, 14, 16 and 18, respectively) [22,23]. These ABAs were fully characterized, especially from a crystallographic point of view, in order to scrutinize the occurrence of dihydrogen H^δ+^···H^δ−^ bonds for the heads of the solid ABAs.

In the work below, the C16AB adduct was selected as a counterpart of C_16_H_33_N(CH_3_)_3_Br and C_18_H_34_COOH. The rationale behind this choice is based on two aspects: (i) the C16AB head NH_2_BH_3_ is expected to interact with NH_3_BH_3_ through dihydrogen H^δ+^···H^δ−^ bonds, and then allow C16AB to act as a soft template for stabilizing AB nanoparticles; (ii) C16AB is made of B, N, C and H only, namely without any other heteroatom (such as Br in cetyltrimethylammonium bromide).

Herein, acetonitrile was used as the solvent and cyclohexane was used as the counter-solvent. Under an argon (≥99.998%) atmosphere and at room temperature, an emulsion was successfully prepared by mixing 506 equivalents of AB with 1 equivalent of C16AB. This emulsion was directly used to prepare the BN/C material. The formed AB@ABA nanostructures first underwent a thermal treatment under autogenous pressure to form oligomers. This inorganic polymerization is the result of dehydrocoupling, as previously described [12]. The thermal decomposition of bulk AB proceeds through a dehydrogenation by a heteropolar dihydrogen interaction between the protonic hydrogen atom (H^δ+^) of the ammonia group (NH_3_) and the hydridic hydrogen (H^δ−^) of the borane moiety (BH_3_). This decomposition occurs through a first-dehydrogenation event at temperatures between 107 and 125 °C (depending on the heating rate), and results in the formation of one equivalent of H_2_ gas along with a BNH_x_ polymeric residue. However, it has been proven that the nanosizing strategy destabilizes AB, thus enabling its thermal decomposition at lower temperatures. Similar effects would take place for the AB@ABA nanostructures, as they are comprised mainly of AB to which an ABA part is added. All the thermal decompositions of the ABAs, studied using a thermogravimetric analysis coupled with gas chromatography and mass spectrometry (TGA-GC-MS), showed that the ABAs decompose comparably to AB (dehydrogenation of the NH_2_BH_3_ group) when heated at 250 °C [24]. This is explained by the inductive effect towards the NH_2_BH_3_ group due to the higher electronegativity of nitrogen in the NH_2_ groups compared to carbon in the CH_x_ groups of the C_x_H_2x+1_ alkyl chain. Meanwhile, the dehydrogenation of C−H bonds in amine borane adducts occurs at much higher temperatures (>200 °C). Consequently, and with consideration of the presence of the carbon group of the ABA, the resulting polymer obtained after the dehydrocoupling step was denoted as the BN(C)H polymer. The advantage of dehydrocoupling under autogenous pressure (in a closed autoclave) is that it offers the possibility to work at lower temperatures. In addition, this method minimizes the weight loss and enhances the homogeneity and size disparity of the material. These BN(C)H-type polymers were then pyrolyzed at temperatures up to 1100 °C to obtain carbon-doped BN (BN/C). The characterization and CO_2_ uptake evaluation of all the BN/C materials are described hereafter.

## 2. Materials and Methods

### 2.1. Material Synthesis

All chemicals were purchased from Merck-Sigma-Aldrich (St. Louis, MO, USA) and used without further purification: a borane dimethyl sulfide (BH_3_-SMe_2_) complex solution (5.0 M in diethyl ether); hexadecylamine; ammonia borane; diethyl ether, DE (anhydrous, ≥99%); tetrahydrofuran, THF (anhydrous, ≥99.9%); acetonitrile, CH_3_CN (ACN); cyclohexane, C_6_H_12_; and boron nitride, BN (1 μm, 98%). Due to the air and moisture sensitivity of the precursors (AB and C16AB), these products were stored and used in an argon-filled glovebox (MBraun Labstar) with an H_2_O and O_2_ content of less than 0.1 ppm.

The C16AB was synthesized according to our previously described procedure [22,25]. In an argon-filled glovebox and at ambient temperature, hexadecylamine (500 mg, 2.07 mmol) was dissolved in 4 mL of diethyl ether. In the glovebox, the borane dimethyl sulfide complex solution (0.46 mL) was slowly added (dropwise) to the amine solution, and the mixture was stirred at 500 rpm for 24 h. In our conditions, a slight excess of the borane reactant was used (1.1 mol for 1.0 mol hexadecylamine). The as-prepared solution was transferred outside the glovebox. The solvent (diethyl ether) and the product (dimethyl sulfide) were extracted by cryo-distillation under a fume hood for 2 h.

The synthesis of the AB@ABA nanostructures is described as follows. After determining the solubility of AB and ABA, as well as the miscibility of several solvents, acetonitrile, CH_3_CN (ACN), and cyclohexane, C_6_H_12_, were selected for the synthesis. The acetonitrile was a solvent for AB and ABA, and the cyclohexane acted as a counter-solvent (immiscible with ACN). This combination allowed the precipitation of AB@ABA nanoparticles, probably at the solvent interface by the formation of reverse micelles. Based on the previous work of Valero-Pedraza et al. [20], who worked on AB nanosizing using cetyltrimethylammonium bromide (CTAB) as the surfactant, a molar ratio of 506 mol of AB to 1 mol of ABA was chosen as a reference, as it enabled the reproducibility of the results. In the case of the adduct C16AB, 0.025 g of it and 1.54 g of AB were solubilized in 0.11 mL and 6.9 mL of ACN, respectively, inside the glovebox. The solutions were then mixed and added dropwise to 21 mL of cyclohexane to form the nanostructures AB@C16AB.

In a typical procedure, the AB@ABA nanostructures were decanted in a Schlenk flask inside the glovebox. The solvent mixture was removed by drying under reduced pressure at 40 °C for 24 h using a Schlenk line. A total of 500 mg of the recovered white solid was transferred to a 45 mL stainless steel autoclave with a PTFE (Teflon)-lined chamber. The autoclave was then sealed, placed in an oven and heated for 72 h at various temperatures (90, 120 and 150 °C). This resulted in a foam-like solid (yield of ~90%).

In the glovebox, the recovered white solid polymer BN(C)H was ground into a fine powder using an agate mortar. About 300 mg were then placed in an alumina crucible and inserted inside a quartz tube. The tube was removed from the glovebox and placed inside a tubular furnace (Carbolite). This tube is a custom-made cylinder recipient that has three inlets/outlets: one connected to a gas line for the gas entry, another connected to the vacuum pump and the last one for the gas exit after it flows through the sample. Before pyrolysis, three reduced-pressure/argon cycles were performed to remove the residual air. The heating process was executed under an argon flow of 50 mL·min^−1^ for 90 min at temperatures up to 1100 °C to obtain carbon-doped BN, noted as BN/C. All BN/C syntheses can be found in Table S1.

### 2.2. Material Characterization

Nitrogen adsorption–desorption at −196 °C using a 3Flex surface analyzer (Micromeritics, Mérignac, France) was used to study the materials’ textural properties. The BN/C samples were degassed at 250 °C for 15 h under reduced pressure (1.33 × 10^−3^ mbar) before the experiment. To determine the specific surface area (SSA) in the range of relative pressure (P/P°) between 10^−5^ and 0.1, the Brunauer–Emmett–Teller model (BET) was applied. The microporous surface area and volume (pore width, w, ≤ 2 nm) were determined using the t-plot model in the linear range of P/P° between approximately 0.2 and 0.5. The total pore volume of the sample was calculated at a relative pressure of 0.99 based on the adsorbed nitrogen volume.

The micropore size distribution was estimated with the non-local density functional theory (NLDFT) calculation method using the SAIEUS software (2.0, Micromeritics, GA, USA).

Scanning electron microscopy (SEM) using a Hitachi S4800 microscope (Hitachi, Buckinghamshire, UK) was used to study the morphology.

The carbon, nitrogen and hydrogen compositions of the optimized BN/C materials were measured using a Vario Micro Cube analyzer (Elementar, Lyon, France). An iCAP 7400 Duo inductively coupled plasma–optical emission spectrometer (ICP-OES, Thermo Scientific, Waltham, MA, USA) was used to determine the boron content. The following wavelengths were analyzed: 249.773, 249.677, 208.959, 182.641 and 136.246 nm. The BN/C material was mineralized before the ICP-OES experiment by dissolution in a mixture of hydrochloric and nitric acids. In a typical procedure, approximately 25 mg of the material was mixed with the acids in an autoclave and heated at 180 °C for 16 h. After cooling down at room temperature, the resulting solution was diluted in water before the analysis. Finally, the O content was determined using SEM-EDX (energy-dispersive X-ray spectroscopy) analyses performed with a Zeiss EVO HD15 electron microscope (Zeiss, Rueil Malmaison, France).

A thermogravimetric analysis (TGA, STA 409 PC Luxx thermal analyzer from NETZSCH, Selb, Germany) using synthetic air was employed to determine the thermal stability. The material (reduced into powder) was placed in an alumina crucible and heated with a ramp of 5 °C·min^−1^ up to 1100 °C.

Powder X-ray diffraction (PXRD) was used to assess the material’s structure. The patterns were recorded on an X’Pert Powder Spinner (PANalytical, Melvern, UK) with a Cu-Kα source (Kα = 0.154 nm).

The PerkinElmer spectrometer Spectrum 2 (PerkinElmer, Waltham, MA, USA), with an attenuated total reflectance (ATR) mode, was used for the FTIR spectroscopy experiments. Nuclear magnetic resonance (NMR) in a solid state for the ^11^B solid was performed on a Varian VNMR4000 spectrometer (Varian, Palo Alto, CA, USA) with a resonance frequency of 128,355 MHz. This apparatus was equipped with a 9.39 T wide-bore magnet using a 3.2 mm magic angle spinning (MAS) probe with a spinning rate of 20 kHz. The standard used as a reference was sodium borohydride with a signal fixed at −42.1 ppm.

### 2.3. CO_2_ Adsorption Measurement

As previously presented for BCN materials [6], CO_2_ uptake measurements using the volumetric method were evaluated with a 3Flex analyzer. Before the analyses, the samples were degassed for 15 h at 250 °C under reduced pressure (1.3 × 10^−3^ mbar) and then measured at 1 bar, at 3 different temperatures (0, 25 and 35 °C). The adsorption uptake is the highest adsorbed quantity, at a given temperature, of the corresponding isotherm located at a pressure of 1 bar. The isosteric adsorption enthalpy was determined using the Clausius–Clapeyron method (Equations (S1) and (S2)) to determine the affinity between an adsorbate and the surface of an adsorbent [26]. We applied this method to plot the isotherms at 0 and 35 °C. Using the ideal adsorption solution theory (IAST, Equation (S3)), we were able to determine the selectivity toward CO_2_ compared to N_2_ [27].

Finally, the recyclability of the BN/C material was tested at 25 °C and 1 bar. The CO_2_ adsorption experiments were performed with the exact same procedure as described earlier and considered as the first cycle uptake. The recyclability percentage was calculated after each cycle (Equation (S4)). It is worth noting that, between each of the 5 cycles undertaken, the sample was degassed under reduced pressure at 150 °C for 2 h.

## 3. Results

### 3.1. Nanostructuration of AB@ABA Emulsion

**Figure 1:**
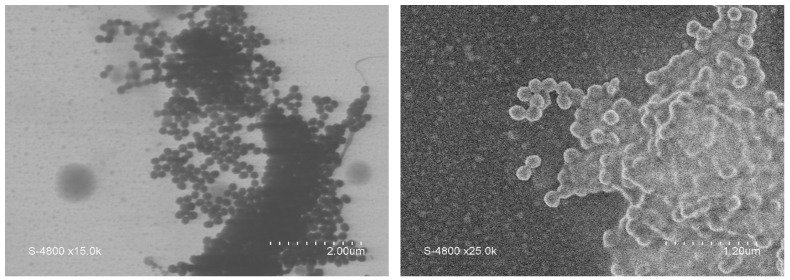
MEB images of AB@ABA emulsion.

The AB@ABA nanostructure (in the ACN/cyclohexane mixture) was mainly characterized by SEM (Figure 1). From these images, we can clearly affirm that spherical nanostructures were obtained. A particle size analysis was carried out using ImageJ software (IJ 1.41, US National Institute of Health, MD, USA), with 200 diameter measurements taken from one image. Figure S1 shows the obtained statistical histogram of the AB@C16AB particle size. The relative narrow size distribution observed was between 120 and 170 nm, and centered at 150 nm. These results have to be compared with early studies of nanostructuring without confining host materials, where sizes of 50 nm (according to Song et al.) [28], from 60 to 120 nm (according to Lai et al.) [29], and averaging 110 nm (according to Valero et al.) were obtained [20]. With the exception of the work of Valero et al., no study has so far demonstrated the possibility of obtaining distinct spherical nanoparticles, but rather, more or less regular nanostructures are obtained.

**Scheme 1:**
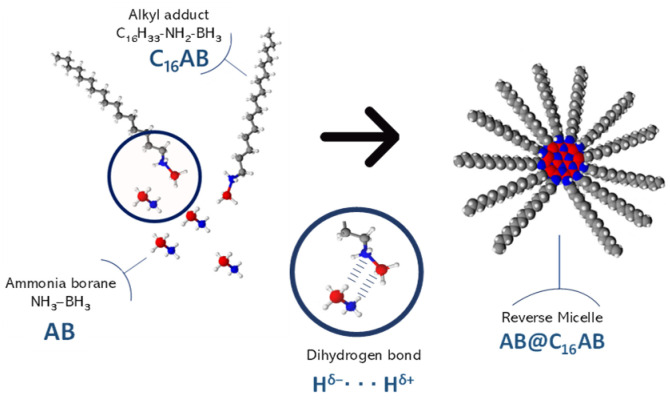
Representation of the nanostructuration driving forces.

From this result, we presume that the spheres are obtained by means of a self-assembly driven by London forces of the C16AB chain length combined with H^δ−^····H^δ+^ interactions within both the AB and the ABA. It is also believed that “reverse micelles” are formed as intermediates to this nanostructuration, as depicted in Scheme 1.

The emulsion was thermally treated directly without further characterization according to the procedure depicted in the Section 2.

### 3.2. Characterization of BN/C

The textural properties of all synthesized BN/C materials (solvent-free and with ACN/cyclohexane mixture) are summarized in Table S1. The corresponding nitrogen adsorption–desorption isotherms are presented in Figure 1 for BN/C-8 and Figure S2 for BN/C1 to BN/C-4. According to Table S2, we can note that the specific surface area (SSA) increased from 172 m^2^·g^−1^ to 585 m^2^·g^−1^ upon increasing the pyrolysis temperature from 600 to 1100 °C (maximal temperature intended, in a solvent-free way). As observed, the microporous surface area and the volume also increased (as seen in the N_2_ sorption isotherms presented in Figure S2). The materials BN/C-1, BN/C-2, BN/C-3 and BN/C-4 presented, according to the IUPAC classification [30], a type I isotherm with different adsorbed N_2_ quantities. This type of isotherm is typical of microporous materials with relatively small external surfaces. They also showed a H4 hysteresis loop, indicating the existence of slit-like pores. The increase in the SSA upon increasing the pyrolysis temperature is explained mainly by the higher release of volatile species (H_2_, C_x_H_y_ and NH_3_) under high temperatures, which leaves more pores in the structure of the BN/C materials. This is often observed in pyrolysis-derived materials such as B-C-N-based materials [6].

The pyrolysis heating rate effect on the textural properties of the “solvent-free” materials was also studied. The isotherms of the corresponding materials are presented in Figure S3. As observed, BN/C-4, BN/C-6 and BN/C-7, with heating rates of 10, 5 and 15 °C·min^−1^, respectively, showed a type I isotherm with a H4 hysteresis loop characteristic of microporous materials. The calculated SSA, according to the BET technique, showed a value of 400 m^2^·g^−1^ for BN/C-6 and 378 m^2^·g^−1^ for BN/C-7. On the contrary, BN/C-5, with a heating rate of 2 °C·min^−1^, presents a type III isotherm typical of non-porous or macroporous materials, with an SSA of only 11 m^2^·g^−1^.

**Figure 2:**
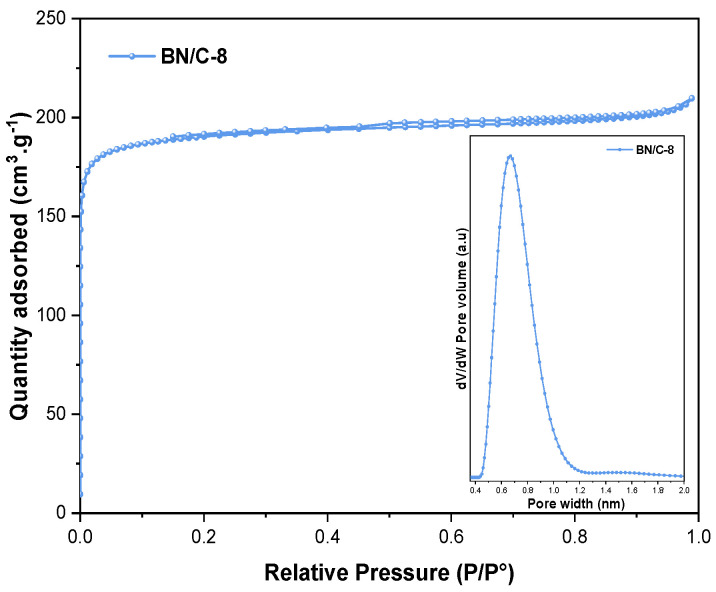
Nitrogen adsorption–desorption isotherm at −196 °C of BN/C-8 (inset: pore size distribution of BN/C-8 by NLDFT method).

Once the pyrolysis heating rate and the temperature were chosen, we studied the solvothermal condition. The emulsion mixture containing the ABA/AB in ACN/cyclohexane was thermally treated without removing the solvents. Figure S3 shows the isotherm of the material prepared using an ACN/cyclohexane volume ratio of 1/3 (i.e., BN/C-8) compared to the solvent-free material (i.e., BN/C-4). BN/C-8 showed (Figure 2) the same type of isotherm as BN/C-4 (type I isotherm with H4 hysteresis loop) with a greater quantity of adsorbed N_2_, suggesting a higher porosity. Indeed, the calculated BET SSA of BN/C-8 reached 767 m^2^·g^−1^ with a pore volume of 0.32 cm^3^·g^−1^. The microporous volume percentage reached 92%. The type of microporosity was detailed by the study of pore size distribution using the NLDFT theory by means of the SAIEUS software. As shown in Figure 2 (inset), BN/C-8 displayed a single peak at 0.67 nm, indicating its pore size monodispersity in the micropore range, and more particularly, its high density of ultra-micropores (w ≤ 0.7 nm). We can clearly note the influence of the synthesis conditions on the N_2_ sorption results, and more specifically, the solvent effect during the polymerization step. We presume the solvent to be trapped during the dehydrocoupling process, creating a cavity within the polymer and, thus, porosity in the final BN/C material.

**Figure 3:**
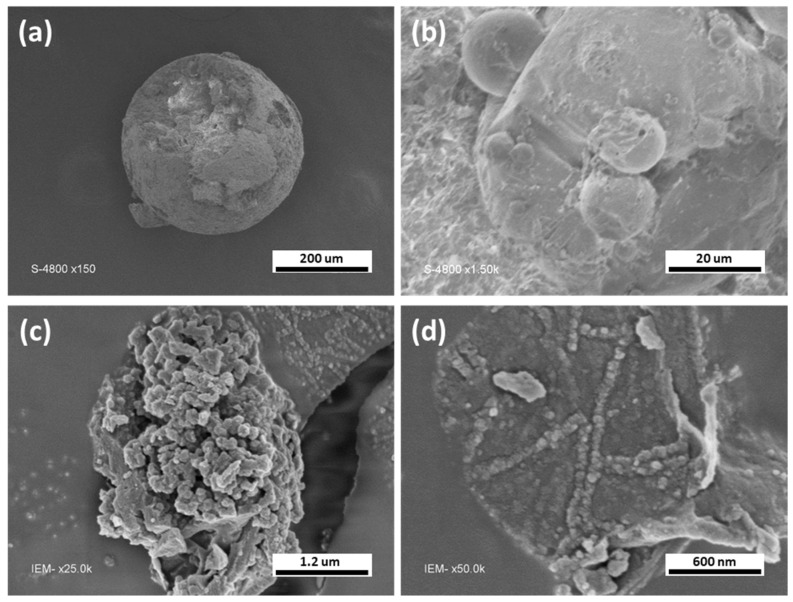
SEM images of BN/C-8 at four magnifications ((**a**): 200 µm; (**b**): 20µm; (**c**): 1.2 µm; (**d**): 600 nm).

This optimized BN/C-8 material was further characterized. The material, and more particularly, the particles’ morphology, was analyzed by SEM. The SEM images of BN/C-8 in Figure 3 generally show a spherical morphology. We observed two distinct sphere sizes: those in the microscale range and those in the nanoscale range. The sphere diameters in the nanoscale range had an average diameter of ca. 39 nm. However, the microscale spheres observed in the SEM images were much fewer, and a particle diameter estimation would not have been accurate.

To quantify the chemical composition of BN/C-8, an elemental analysis was performed for C, N and H. The weight percentages of each element are displayed in Table 1, indicating an atomic ratio of B_1_C_0.03_N_0.92_H_0.57_O_0.39_. The amount of C was much lower than those of B and N due to the initial molar ratio for AB/C16AB of 506/1.

Although this quantification was performed under inert conditions (Ar atmosphere), we observed a certain amount of oxygen. This might have been due to equipment-sealing defaults and the small water content even in anhydrous solvents.

**Figure 4:**
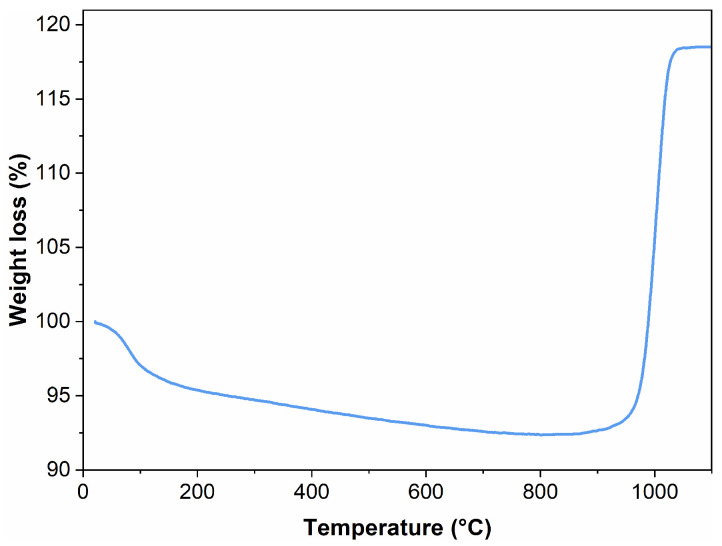
TGA curve of BN/C-8 (in air, 5 °C·min^−1^).

The weight variation of BN/C-8 upon heating up to 1100 °C at 5 °C·min^−1^ under an air flow (20% O_2_, 80% N_2_) of 50 mL·min^−1^ is depicted in Figure 4. BN/C-8 gradually lost 8% of its mass when the temperature was increased up to 900 °C. This low weight loss is ascribed to carbon oxidation [31,32] (1.2 wt%) as well as the partial nitrogen oxidation, resulting in the release of nitrogen oxide [33]. On the other hand, the weight increase above 900 °C, which reached 26%, is attributed to unstable boron moiety oxidation, forming boron oxide [34]. Overall, the BN/C-8 material showed a good thermal stability and a high oxidation resistance up to 900 °C.

**Figure 5:**
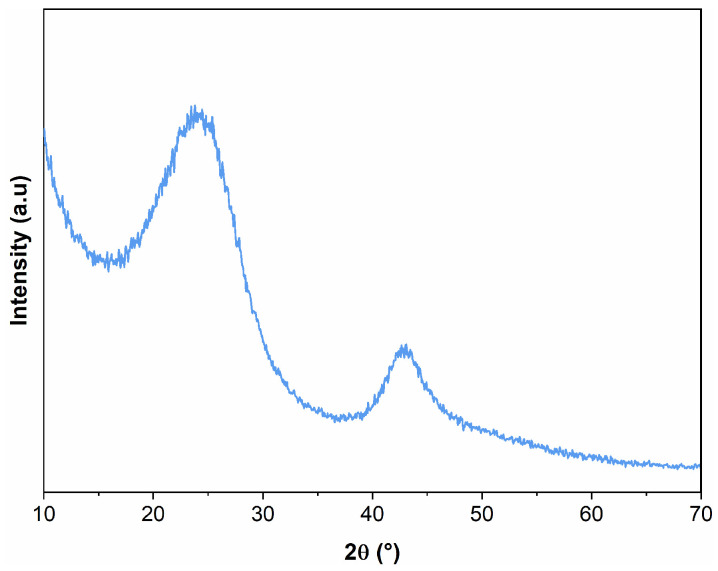
PXRD pattern of BN/C-8.

The PXRD pattern of the BN/C-8 material, depicted in Figure 5, shows two broad diffraction peaks. The first peak is centered at 24.1° and the second is centered at 42.9°. The peaks correspond to the (002) and (100) reflection planes, which are characteristics of both hexagonal borocarbonitride (ICDD 00-035-1292) and hexagonal boron nitride (ICDD 00-009-0012). In comparison with crystalline h-BN, the BN/C-8 material showed a general broadening of diffraction planes and a shift of the (002) plane towards lower 2θ values (located at 26.7° for h-BN), thereby indicating that the doping of carbon may have produced structural defects and an increase in the distance between the material layers [35,36,37].

The interlayer distance in the first-order diffraction (n = 1) according to the (100) reflection plane, denoted by d_100_, is estimated to be 0.21 nm, which is similar to h-BN. However, the d_002_ (0.37 nm) is higher than the d_002_ of h-BN, which is equal to 0.33 nm according to the literature [38].

**Figure 6:**
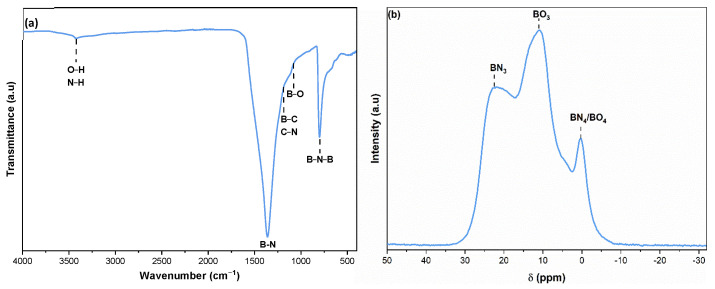
(**a**) FTIR spectrum of BN/C-8; (**b**) ^11^B MAS NMR spectrum of BN/C-8.

The FTIR spectrum of BN/C, depicted in Figure 6a, showed two main bands centered at 1367 cm^−1^ and 792 cm^−1^, corresponding to the in-plane stretching vibration of *B−N* and to the *B−N−B* bending vibration, respectively. The broad band at 3407 cm^−1^ is attributed to the *O−H* and/or *N−H* vibration bonds. The small shoulder band detected at 1078 cm^−1^ is attributed to the *B−O* stretching vibration of tetragonal BO_4_ groups. Finally, another very small shoulder band was detected at 1178 cm^−1^ and could designate the stretching vibration of *C−N* and/or *B−C* bonds, which are usually located between 1100 cm^−1^ and 1300 cm^−1^ [35,39,40,41]. Note that a stretching vibration of the *C=N* bond, located around 1600 cm^−1^, is usually detected in *B−C−N* materials, but was not detected in the obtained FTIR spectra, probably due to the overlap with the huge *B−N* band [42].

It is worth mentioning that an asymmetric stretching vibration of trigonal BO_3_ groups is usually detected in the range between 1200 and 1500 cm^−1^, which, in this case, may have overlapped with that of *B−N*.

The chemical environment of the B atoms was assessed using solid-state ^11^B MAS NMR spectroscopy. The spectrum in Figure 6b shows three distinct signals. The first signal at 22.4 ppm was assigned to the B-N bond of the planar BN_3_ groups [31,43,44]. The peak at 11.1 ppm can be attributed to trigonal BO_3_ and BN_2_H functions [31,43]. For the peak at 0.8 ppm, several suggestions have been reported in the literature. It is often assigned to tetragonal BN_4_ [44] groups or tetragonal BO_4_ groups [31]. It is difficult to conclude among these possibilities, but we presume that it was the result of a combination of tetragonal BN_4_ and BO_4_ groups.

### 3.3. CO_2_ Adsorption Measurement

**Figure 7:**
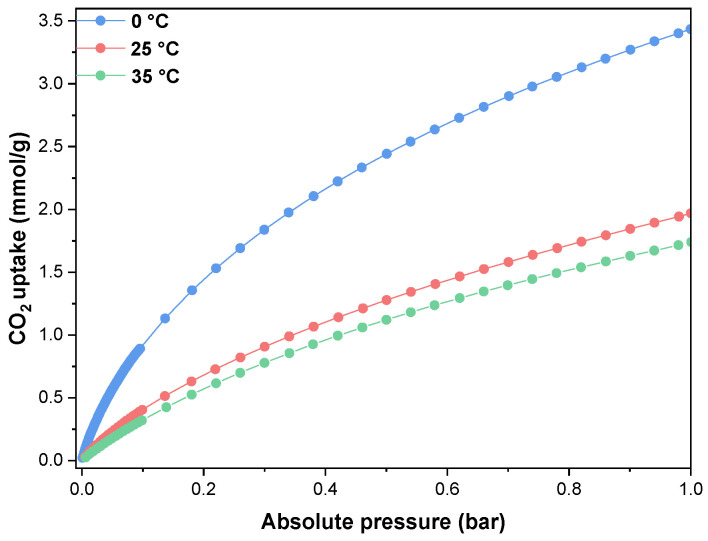
CO_2_ uptake by BN/C-8 at 0 °C, 25 °C and 35 °C (up to 1 bar).

The CO_2_ uptake of BN/C-8 at 0, 25 and 35 °C and an absolute pressure of up to 1 bar are represented in Figure 7. A commercial macroporous BN with an SSA of 13 m^2^·g^−1^ was also analyzed to study the effect of porosity and carbon-doping. The main results are summarized in Table 2.

BN/C-8 showed a much higher CO_2_ uptake (3.43 mmol·g^−1^ or 151 mg·g^−1^ at 0 °C) than commercial BN (0.09 mmol·g^−1^ or 4 mg·g^−1^ at 0 °C), which mainly evidences the effect of the porosity on the adsorption uptake. As previously described, the presence of ultra-micropores has been proven to enhance CO_2_ adsorption. This is due to the compatibility of the size of these ultra-micropores (<0.7 nm) with the CO_2_ kinetic diameter. This performance could further account for the carbon-doping effect, as it is an enhancing factor for CO_2_ uptake. Indeed, it has been proven that carbon-doping alters the surface chemistry of boron nitrides and creates structural defects, which increases the number of active sites such as basic N groups suitable for acidic CO_2_ adsorption. From these results, we can also notice that the CO_2_ uptake of the BN/C-8 materials decreased upon increasing the adsorption temperature. This is a typical behavior, as the adsorption is a spontaneous exothermic process [45,46].

**Figure 8:**
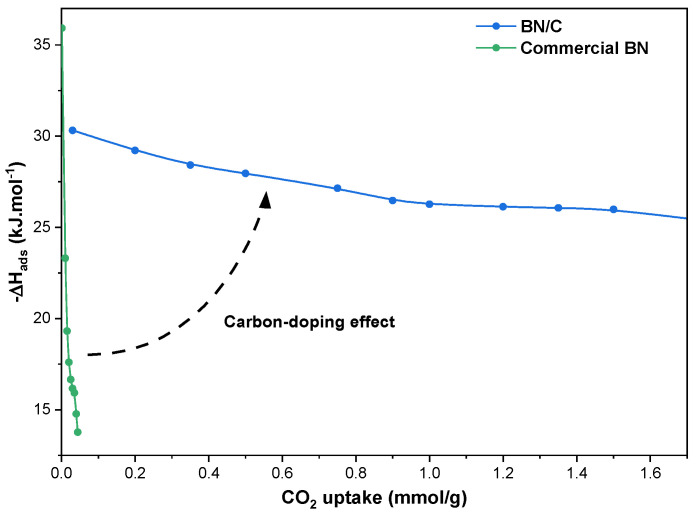
Isosteric adsorption enthalpies of BN/C-8 and commercial BN as a function of the quantity of adsorbed CO_2_.

To evaluate the interaction between CO_2_ and the surface of the substrate, we determined the isosteric enthalpy of adsorption, $\Delta H_{\mathrm{ads}}$. As shown in Figure 8, the BN/C-8 material showed adsorption enthalpies in the range of −25 to −30 kJ·mol^−1^ for CO_2_ uptake values between 0.05 and 1.74 mmol·g^−1^. These values fit well in the range of physisorption (>−50 kJ·mol^−1^), and they show that the CO_2_ interactions with the BN/C-8 material are mainly governed by physical weak interactions and that no (or very little) chemisorption (<−60 kJ·mol^−1^) took place.

Commercial BN generally presented higher enthalpies of adsorption (which means a lower isosteric heat of adsorption) ranging between −14 and −36 kJ·mol^−1^, indicating that it forms weaker interactions with CO_2_ than the BN/C-8 material. Tian et al. also reported a CO_2_ adsorption uptake for BN of 0.13 mmol·g^−1^ (at 25 °C and 1 bar), which reached 0.95 mmol·g^−1^ after carbon-doping. Their study was supported by DFT calculations [47].

According to Figure 8, the adsorption enthalpy appears higher for low CO_2_ uptake values and then decreases with an increase in the surface coverage. This demonstrates the heterogeneity of the material’s surface and a better diffusion of CO_2_ in narrow pores [48].

**Figure 9:**
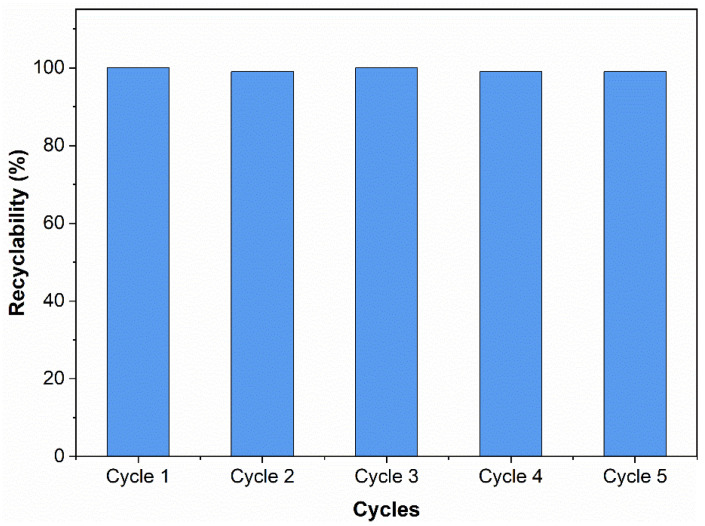
CO_2_ recyclability at 25 °C of BN/C-8.

CO_2_ adsorption recyclability tests were performed to evaluate the sustainability of the BN/C material. Figure 9 shows the material’s CO_2_ uptake after five adsorption–desorption cycles. As can be seen, BN/C-8 had an outstanding recyclability with a recycling percentage of 99% after five cycles. The ease of recyclability of the BN/C-8 material is explained by the dominant physical nature of the adsorbent–adsorbate interactions, in accordance with the aforementioned isosteric adsorption enthalpy values.

**Figure 10:**
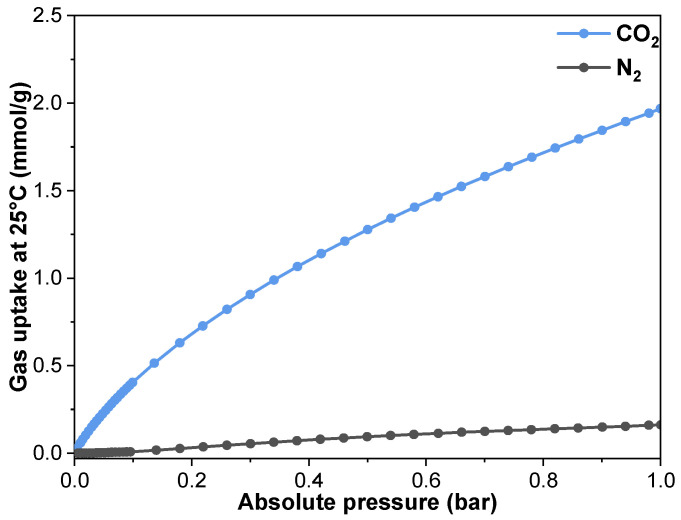
CO_2_ and N_2_ adsorption isotherms at 25 °C of BN/C-8.

The potential of a material to selectively adsorb CO_2_ as opposed to N_2_ is an important factor for both air and flue gas decarbonization applications. Figure 10 shows the adsorption isotherms of N_2_ and CO_2_ at 25 °C. We noticed that the CO_2_ uptake values were much higher than those of N_2_ in any pressure range. This was confirmed by the selectivity value obtained using the IAST method for a flue gas composition, which was found to be 21.

As described, this carbon-doped BN material presents a high surface area, but also a good affinity for CO_2_. Our synthesized material presents a CO_2_ adsorption uptake of up to 3.43 mmol·g^−1^ at 0 °C, with a great CO_2_/N_2_ selectivity (21).

The structural defects induced in the BN structure by the carbon-doping had a positive influence on the CO_2_ sorption, as already shown by Tian et al. [47] and Chen et al. [43].

In addition, this type of porous material was obtained in two steps, using only boron, carbon, nitrogen and hydrogen elements, without a porosity agent. Furthermore, we proved that the nanostructures obtained in the emulsion state remained after heat treatments. Finally, as demonstrated by the TGA experiment, these nanostructures are thermally stable and resistant to oxidation.

## 4. Conclusions

A porous BN/C material was synthesized from a nanostructured mixture of a hexadecyl amine borane adduct and ammonia borane (AB@ABA) without using a porosity agent. The synthesis integrated two main steps, with the first one being the synthesis of the nanostructured mixture and the second one consisting of the thermolysis (dehydrocoupling followed by pyrolysis) of the mixture. The BN/C material maintained its nanostructure after pyrolysis, as depicted by SEM experiments. This material is mainly microporous and has a specific surface area of up to 767 m^2^·g^−1^ and a pore volume of 0.32 cm^3^·g^−1^. As shown by the N_2_ and CO_2_ adsorption analyses, the porosity of this BN/C material is accessible, and the pore size of the synthesized nanostructured BN/C material offers a good potential for CO_2_ capture. We indeed demonstrated a CO_2_ uptake of up to 3.43 mmol·g^−1^ at 0 °C. According to the IAST method, a significant CO_2_/N_2_ selectivity value of 26 was also proven. Further works will be undertaken to study other AB/C16AB content as well as other ABA candidates that might improve the adsorption capacities.

## Figures and Tables

**Table 1:** Chemical composition and atomic ratios of BN/C-8.

Material	B wt%	C wt%	N wt%	H wt%	O wt%	Atomic Ratio
BN/C	35.00 ±0.20	1.20 ±0.24	41.50 ±0.17	1.80 ±0.25	19.9	B_1_C_0.03_N_0.92_H_0.57_O_0.39_

**Table 2:** CO_2_ uptake performances of BN/C-8 and commercial BN at 1 bar.

Sample	CO_2_ Uptake (mmol·g^−1^) (in mg·g^−1^)			CO_2_/N_2_ IAST Selectivity	Recyclability after 5 Cycles
	0 °C	25 °C	35 °C		
BN/C	3.43 (151)	1.97 (87)	1.74 (77)	21	99%
Commercial BN	0.09 (4)	0.04 (2)	-	-	-

## Data Availability

Data sharing is not applicable to this article.

## Supplementary Materials

The following supporting information can be downloaded at: https://www.mdpi.com/article/10.3390/nano13172389/s1, Table S1: The synthesis conditions of the BN/C materials prepared from the AB@C16AB nanostructure; Table S2: Textural properties of the BN/C materials prepared from the AB@C16AB nanostructure; Figure S1: Statistical histogram of AB@ABA particle size from ImageJ software; Figure S2: Nitrogen adsorption–desorption isotherms at −196 °C of BN/C-1 to BN/C-4; Figure S3: Nitrogen adsorption–desorption isotherms at −196 °C of BNC-4 to BN/C-7 using an ACN/cyclohexane mixture; Equation (S1): Clausius–Clapeyron equation; Equation (S2): Equation obtained by solving Equation (S1); Equation (S3): Ideal adsorption solution theory; Equation (S4): Recyclability calculation of CO_2_ uptake after 5 cycles.
