# Supplementary material for: Nanostructured Carbon-Doped BN for CO2 Capture Applications
Source: Nanomaterials (Basel). 2023 Aug 22;13(17):2389. doi: 10.3390/nano13172389 (PMC10490533; doi:10.3390/nano13172389)
Supplement: Supplementary file 1 [file nanomaterials-13-02389-s001.zip › nanomaterials-2511034-supplementary.pdf]

Supplementary Materials

Nanostructured Carbon Doped BN for CO_2 Capture Applications

Rimeh Mighri[1], Kevin Turani-I-Belloto[2], Umit B. Demirci[2] and Johan G. Alauzun[1*]

[1] Institut Charles Gerhardt, Univ Montpellier, CNRS, ENSCM, Montpellier, France
[2] Institut Europeen des Membranes, IEM – UMR 5635, Univ Montpellier, ENSCM, CNRS, Montpellier, France
* johan.alauzun@umontpellier.fr.

Equation S1: Clausius–Clapeyron

$$\Delta H_{ads} = R\left[\frac{\partial \ln P}{\partial\left(\frac{1}{T}\right)}\right]_q \quad (S1)$$

In this equation, $[\Delta H]_{ads}$ corresponds to the isosteric adsorption enthalpy (kJ.mol^{-1}), R to the ideal gas constant (8.314 J.mol^{-1}.K^{-1}) and q to the adsorbed quantity of CO_2 at a pressure P and temperature T.

Equation S2: Obtained by solving equation S1

$$\Delta H_{ads} = -\left(\frac{RT_1T_2}{T_2 - T_1}\ln\frac{P_2}{P_1}\right) \; (S2)$$

Equation 3: Ideal Adsorption Solution Theory (IAST)

$$S_{CO_2/N_2} = \frac{q_1/q_2}{P_1/P_2} \quad (S3)$$

q_1 and q_2 are respectively the adsorbed quantity of CO_2 and N_2 at P_1 and P_2, P_1 = 0.15 bar and P_2 = 0.75 bar.

Equation S4: Recyclability calculation of CO_2 uptake after 5 cycles.

$$Recyclability\,(\%) = \frac{Qads_{Cycle\,5}}{Qads_{Cycle\,1}} * 100 \quad (S4)$$

Table S1. The synthesis conditions of the BN/C materials prepared from the AB@C16AB nanostructure.

Material	Varied conditions	Solvent	ACN / cyclohexane volume ratio	Polymerization T (°C)	Pyrolysis T (°C)	Heating rate (°C/min)
**BN/C-1**	**Pyrolysis temperature**	**Solvent-free**	**-**	**90**	600	10
**BN/C-2**		Solvent-free	-	90	800	10
**BN/C-3**		Solvent-free	-	90	1000	10
**BN/C-4**		Solvent-free	-	90	1100	10
**BN/C-5**	Pyrolysis heating rate	Solvent-free	-	90	1100	2
**BN/C-6**		Solvent-free	-	90	1100	5
**BN/C-7**		Solvent-free	-	90	1100	15
**BN/C-8**	Polymerization with solvent	ACN / cyclohexane	1/3	90	1100	10

Table S2. Textural properties of the BN/C materials prepared from the AB@C16AB nanostructure.

Material	BET SSA		Pore volume	
	Total SSA (m^2/g)	t-plot SSA (%)	Total V (cm^3/g)	t-plot V (%)
**BN/C-1**	172	63	0.13	50
**BN/C-2**	235	88	0.15	61
**BN/C-3**	322	94	0.21	66
**BN/C-4**	585	94	0.32	67
**BN/C-5**	11	-	0.09	-
**BN/C-6**	400	87	0.33	42
**BN/C-7**	378	82	0.30	47
**BN/C-8**	767	98	0.32	92

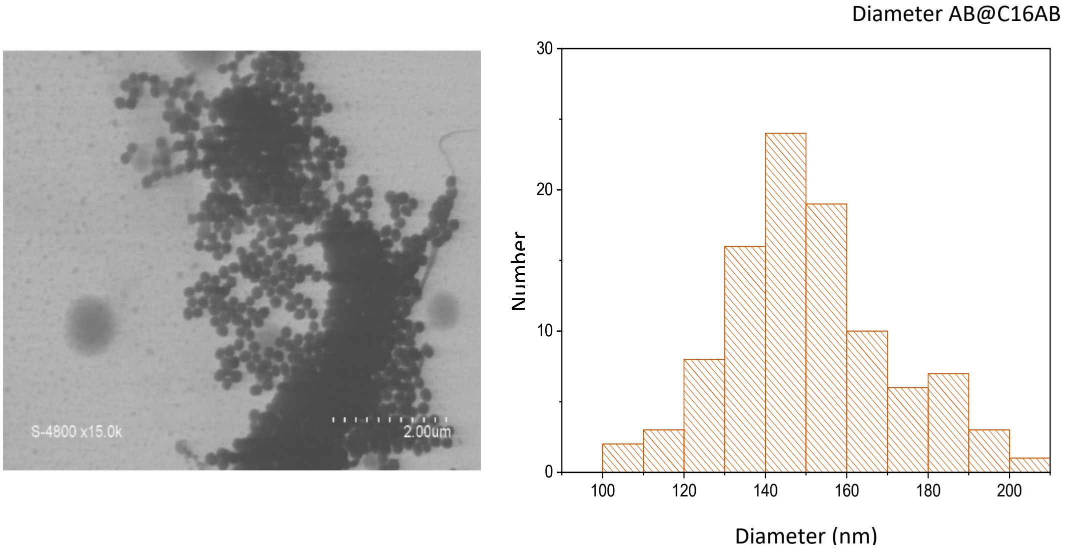

Figure S1. Statistical histogram of AB@C16AB particle size from ImageJ software.

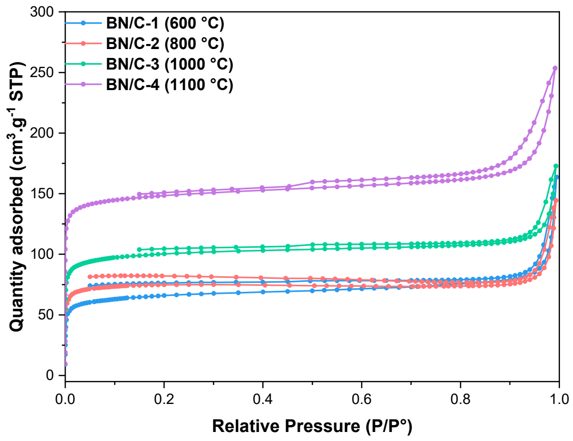

Figure S2. Nitrogen adsorption-desorption isotherms at -196 °C of BN/C-1 to BN/C-4.

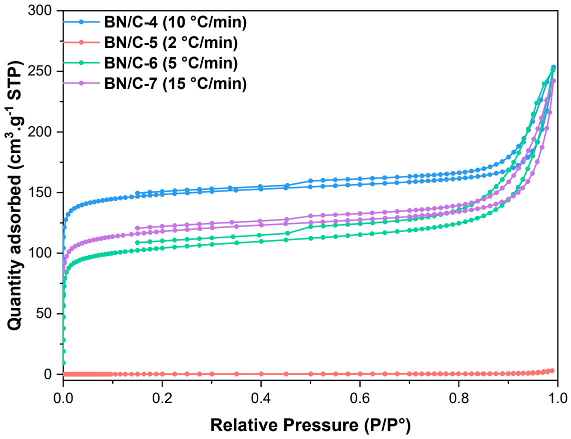

Figure S3. Nitrogen adsorption-desorption isotherms at -196 °C of BNC-4 to BN/C-7 using an ACN/cyclohexane mixture.
